# Characterisation of drug-resistant *Mycobacterium tuberculosis* mutations and transmission in Pakistan

**DOI:** 10.1038/s41598-022-11795-4

**Published:** 2022-05-11

**Authors:** Gary Napier, Anwar Sheed Khan, Abdul Jabbar, Muhammad Tahir Khan, Sajid Ali, Muhammad Qasim, Noor Mohammad, Rumina Hasan, Zahra Hasan, Susana Campino, Sajjad Ahmad, Baharullah Khattak, Simon J. Waddell, Taj Ali Khan, Jody E. Phelan, Taane G. Clark

**Affiliations:** 1 Faculty of Infectious and Tropical Diseases, London School of Hygiene and Tropical Medicine, London, UK; 2 Department of Microbiology, Kohat University of Science and Technology, Kohat, Pakistan; 3 Laboratory Hayatabad Medical Complex, Provincial Tuberculosis Reference, Peshawar, Pakistan; 4 Department of Medical Lab Technology, University of Haripur, Haripur, Pakistan; 5 Institute of Molecular Biology and Biotechnology, The University of Lahore, KM Defence Road, Lahore, 58810 Pakistan; 6 Institute of Biotechnology and Microbiology, Bacha Khan University, Charsadda, Pakistan; 7 Department of Pathology and Laboratory Medicine, The Aga Khan University, Karachi, Pakistan; 8 Institute of Institute of Pathology and Diagnostic Medicine, Khyber Medical University, Peshawar, Khyber Pakhtunkhwa Pakistan; 9 Global Health and Infection, Brighton & Sussex Medical School, University of Sussex, Falmer, BN1 9PX UK; 10 Faculty of Epidemiology and Population Health, London School of Hygiene and Tropical Medicine, London, UK

**Keywords:** Genomics, Microbial genetics

## Abstract

Tuberculosis, caused by *Mycobacterium tuberculosis*, is a high-burden disease in Pakistan, with multi-drug (MDR) and extensive-drug (XDR) resistance, complicating infection control. Whole genome sequencing (WGS) of *M. tuberculosis* is being used to infer lineages (strain-types), drug resistance mutations, and transmission patterns—all informing infection control and clinical decision making. Here we analyse WGS data on 535 *M. tuberculosis* isolates sourced across Pakistan between years 2003 and 2020, to understand the circulating strain-types and mutations related to 12 anti-TB drugs, as well as identify transmission clusters. Most isolates belonged to lineage 3 (n = 397; 74.2%) strain-types, and were MDR (n = 328; 61.3%) and (pre-)XDR (n = 113; 21.1%). By inferring close genomic relatedness between isolates (< 10-SNPs difference), there was evidence of *M. tuberculosis* transmission, with 55 clusters formed consisting of a total of 169 isolates. Three clusters consist of *M. tuberculosis* that are similar to isolates found outside of Pakistan. A genome-wide association analysis comparing ‘transmitted’ and ‘non-transmitted’ isolate groups, revealed the *nusG* gene as most significantly associated with a potential transmissible phenotype (P = 5.8 × 10^–10^). Overall, our study provides important insights into *M. tuberculosis* genetic diversity and transmission in Pakistan, including providing information on circulating drug resistance mutations for monitoring activities and clinical decision making.

## Introduction

Tuberculosis disease (TB), caused by bacteria in the *Mycobacterium tuberculosis* complex, is a major global public health problem. Pakistan is a high-burden TB country, being one of eight countries accounting for two-thirds of the estimated 10 million people globally that fell ill with the disease^1^. In 2019, Pakistan had ~ 570,000 TB cases (incidence rate 263 per 100,000) and 43,900 deaths^1^, but disease control is being compromised by increasing HIV prevalence and drug resistance. The country has a high burden for rifampacin resistant (RR-TB), as well as multidrug-resistance (MDR-TB), which is the additional resistance to isoniazid treatments. Pre-extensive drug resistance (pre-XDR-TB) is prevalent^1,2^, involving *M. tuberculosis* that are MDR-TB and resistant to any fluoroquinolone or at least one of the three second-line injectable drugs (capreomycin, kanamycin, amikacin). XDR-TB requires resistance to any fluoroquinolone and a second-line injectable. In January 2021, WHO updated these definitions of XDR-TB to include other drugs, such as bedaquiline^3^. Here, we adopt the older version of the definition as the underlying cases were treated within that framework. There were ~ 25,000 cases of MDR-/RR-TB in 2019^1^. The National TB control program aims to reduce by half the prevalence of TB in the general population by 2025, but to achieve this will require the scaling-up of TB detection and clinical care, as well as improved systems for inferring disease transmission, thereby facilitating further targeted interventions.

Whole genome sequencing (WGS) is revolutionizing our understanding of drug resistance and clinical management, as well as transmission patterns, thereby assisting disease control^4^. *M. tuberculosis* drug resistance is linked to genomic variants in drug targets or pro-drug activators, including single nucleotide polymorphisms (SNPs) and small insertions and deletions (indels), some occurring in gene–gene interactions. It is therefore possible to predict resistance genotypically for 19 anti-TB drugs and their groups (e.g. floroquinolines) using curated libraries of > 1000 mutations across > 30 loci^5,6^, thereby personalizing treatment. Genotypic predictions are an alternative to bacterial culture-based phenotypic drug susceptibility testing (DST), which can be time-consuming and resource intensive, with reproducibility and inhibitory concentration cut-off challenges for particular drugs^5^. Further, WGS data infers the population structure within the *M. tuberculosis* complex, which is phylo-geographical in nature, with strains falling within distinct (sub-)lineages^7^, and potential transmission chains identified through isolates with (near-)identical genomic variation^8^. The identification of highly virulent strain-types or lineages, drug resistance, and transmission clusters will assist the targeting of limited resources for TB control.

There have been recent studies using WGS to characterize *M. tuberculosis* genetic diversity in isolates sourced from Pakistan, where the predominant strains are from the Central Asian (CAS) family, set within lineage 3^2,9–13^. A recent study of TB endemic province of Khyber Pakhtunkhwa (North West Pakistan) found that known mutations in *rpoB* (e.g. S405L), *katG* (e.g. S315T), or *inhA* promoter loci explain the majority of MDR-TB, but there was evidence of complex mixed infections and heteroresistance, which may reflect the high transmission nature of the setting^13^. An earlier study in the same province found similar MDR-TB mutations, but also additional variants in genes conferring resistance to other first and second-line drugs, including in *pncA* (pyrazinamide), *embB* (ethambutol), *gyrA* (fluoroquinolones), *rrs* (aminoglycosides), *rpsL, rrs* and *gid* (streptomycin) loci. Further, acquisition of rifampicin resistance often preceded isoniazid in these isolates, and a high proportion (~ 18%) of pre-MDR isolates had fluoroquinolone resistance markers, being a class of antibiotics that is widely available and used^2^. Eighteen *M. tuberculosis* isolates clustered within eight networks, thereby providing evidence of drug-resistant TB transmission in the Khyber Pakhtunkhwa province^2^. An investigation of XDR-TB isolates sourced across four provinces in Pakistan found similar genes linked to drug resistance as in Khyber Pakhtunkhwa^11^, and an increased frequency and expression of novel SNP mutations in efflux pump genes, potentially explaining some drug resistance^11^.

Here, we analyse 535 M*. tuberculosis* samples with WGS data, collected between years 2003 and 2020, with phenotypic testing of resistance across 12 drugs (rifampicin, isoniazid, ethambutol, pyrazinamide, streptomycin, ofloxacin, moxifloxacin, amikacin, kanamycin, capreomycin, ciprofloxacin, ethionamide). By identifying ~ 38 k SNPs, and inferring genotypic drug resistance across 19 anti-TB drugs (as well as fluoroquinolone and aminoglycoside classes), we sought to understand the phylogeny of *M. tuberculosis* in the largest Pakistan dataset, identify transmission events, and infer commonly circulating mutations linked to drug resistance. The genetic insights were validated in a large *M. tuberculosis* collection (n = 34 k) with WGS and drug susceptibility test data^7^.

## Results

### Isolates and whole genome sequencing data

A total of 535 M*. tuberculosis* isolates sourced between years 2003 and 2020 from Pakistan with publically available WGS and phenotypic susceptibility testing were analysed^2,9–13^. These isolates covered at least four provinces (Balochistan, Khyber Pakhtunkhwa, Punjab, Sindh), but a high proportion of locations were missing (69.5%), all from one study^12^ (Table 1). The majority of samples were from lineage 3 (L3 397, 74.2%; CAS strains), but the other main lineages were represented (L4, 80, 15.0%, including LAM, T and X strains; L2 36, 6.7%, including Beijing; L1 22, 4.1%) (Table 1; S1 Table)**.**

**Table 1:** *Mycobacterium tuberculosis* samples (N = 535).

Characteristic	Group	N	%
Lineage	1	22	4.1
	2	36	6.7
	3	397	74.2
	4	80	15.0
Drug resistance status^a^	Sensitive	60	11.2
	Pre-MDR	31	5.8
	MDR	328	61.3
	Pre-XDR	47	8.8
	XDR	66	12.3
	Other	3	0.6
Individual drug resistance^a^	Rifampicin	460	86.0
	Isoniazid	435	81.3
	Ethambutol	385	72.0
	Pyrazinamide	258	48.2
	Streptomycin	238	44.5
	Ofloxacin	277	51.8
	Moxifloxacin	277	51.8
	Levofloxacin	277	51.8
	Amikacin	75	14.0
	Kanamycin	79	14.8
	Capreomycin	78	14.6
	Ciprofloxacin	277	51.8
	Ethionamide	102	19.1
	Para aminosalicylic acid	10	1.9
	Cycloserine	2	0.4
	Clofazimine	1	0.2
	Bedaquiline	1	0.2
	Fluoroquinolones	277	51.8
	Aminoglycosides	75	14.0
Collection year	2003—2005	49	9.2
	2015—2017	438	81.9
	2018—2020	48	9.0
Region	Peshawar	77	14.4
	Dera Ismail Khan	25	4.7
	Abbottabad	13	2.4
	Swat	13	2.4
	Rawalpindi	7	1.3
	Hyderabad	5	0.9
	Karachi	5	0.9
	Lahore	5	0.9
	Other	13	2.4
	Missing	372	69.5

^a^Genotypic prediction using TB-Profiler.

As expected phenotypic drug susceptibility testing (DST) was performed most often for first-line rifampicin (n = 487, 91.0%), isoniazid (n = 487, 91.0%), ethambutol (n = 479, 89.5%), and pyrazinamide (n = 444, 83.0%) (S2 Table). A total of 432 samples (80.7%) were phenotypically resistant to at least one drug (median 3, maximum 10). The number of potential errors on the phenotypic testing appeared modest (218/2430 tests, 9.0%), where established genotypic resistance markers were present in isolates with DST results that implied drug susceptibility. The discordance appeared for nine drugs, but more than half occurred in two drugs (ethambutol 96; pyrazinamide 42) (S2 Table). The majority of isolates were genotypically assessed as MDR-TB (328, 61.3%), with proportions of (pre-) XDR (113, 21.1%) and pan-sensitive (60, 11.2%) (Table 1). There were 31 pre-MDR isolates, and overall there was a high prevalence of rifampicin (460, 86.0%) and isoniazid (435, 81.3%) resistance associated mutations. Resistance to other drugs was also detected, including ethambutol (385, 72.0%), pyrazinamide (258, 48.2%), streptomycin (238, 44.5%), ethionamide (102, 19.1%), any fluoroquinolone (277, 51.8%) or aminoglycoside (75, 14.0%). Very few isolates appeared resistant to bedaquiliine, clofazimine and cycloserine (n < 3; Table 1). Across all lineages, the majority of isolates (> 75%) were at least MDR-TB resistant (S3 Table).

**Figure 1:**
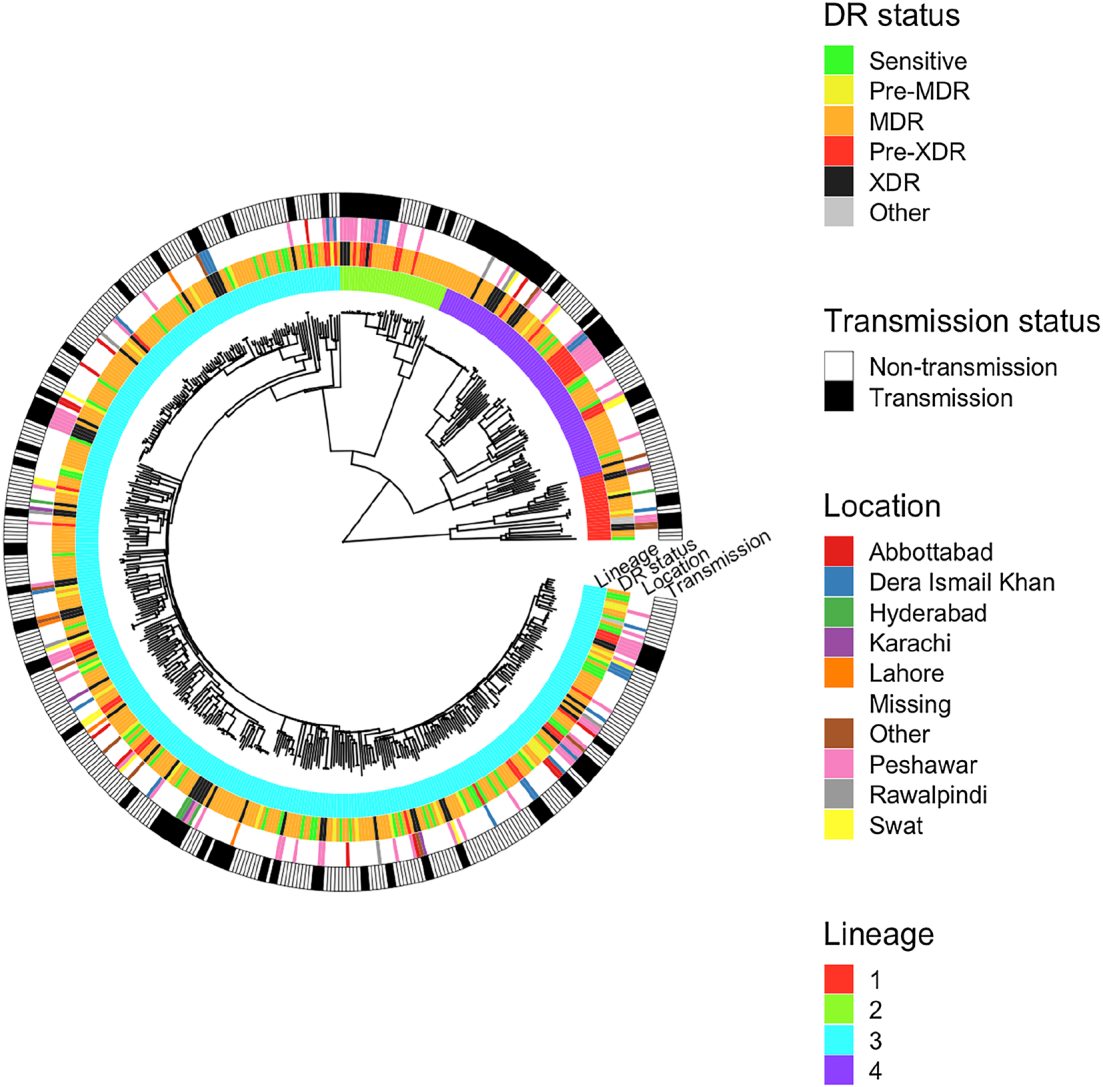
A phylogenetic tree for the 535 M*. tuberculosis* isolates constructed using 37,970 SNPs. The surrounding rings of data for each isolate include: lineage (inner), drug resistance status, location, and transmission status (outer).

After sequence data alignment, high average coverage was observed across the samples (median 76-fold, range 30—2027 fold). Across the isolates, a total of 37,970 genome-wide SNPs were identified, with 23,741 (62.5%) found in single samples. A phylogenetic tree constructed using the 37,970 genome-wide SNPs revealed the expected clustering by lineage (Fig. 1; S1 Figure).

### Evidence of transmission

The median (range) pairwise SNP differences across the 535 isolates was 390 (minimum 0, maximum 1811), with a multi-modal distribution, where modes represent differences within and between lineages (S2 Figure). At a threshold of 10 SNPs, 55 clusters formed consisting of a total of 169 isolates, where the median number of isolates in each cluster was 2 (range: 2—22) (S2 Figure). By reducing the cut-off to 5 SNPs, there were only 6 less clusters (total 49) consisting of a total of 33 isolates (overall 136 isolates) (S4 Table). The 169 transmitted isolates (SNP cut-off 10) were found in three of the four provinces recorded (Khyber Pakhtunkhwa 71/169; Punjab 9/169; Sindh 9/169), identified across all lineages (L1 7/169, L2 21/169, L3 98/169, L4 43/169) and in (pre-)XDR (75/169) samples (S3 Figure; S4 Figure). Most clusters had samples with the same drug resistance phenotype (44/55), and there was some evidence of clusters consisting of more than one location (35/55, excluding missing locations) (S3 Figure; S4 Figure). Comparing the 169 "transmitted" isolates in clusters to the others ("non-transmitted"; n = 366), there were overall differences in lineage (Chi-Square, P < 6 × 10^–8^) and drug resistance (Chi-square P < 5 × 10^–15^). Specifically, there was marginally weak evidence of an increased risk of transmission in lineage 2 (odds ratio (OR) = 3.00, P = 0.054) and lineage 4 (OR = 2.49, P = 0.073), compared to lineage 1. Signals of increased risk of transmission were stronger among those pre-XDR/XDR (OR = 5.79, P < 5 × 10^–14^), compared to a less resistant status. There was no association between transmission risk and province (Chi-Square P = 0.64), but there were high levels of missing location data (S5 Table).

A genome-wide association study (GWAS) approach was applied to detect loci potentially linked to transmissibility. It revealed *nusG, Rv2307B, wag31, proX* and *murA* genes to be the most associated with being in a transmission cluster (P < 10^–5^) (S6 Table). *Rv2307* (beta = 0.745, P = 1.5 × 10^–8^) putatively codes for a glycine rich protein, while *proX* (beta = 0.706, P = 1.3 × 10^–6^) encodes osmoprotectant binding lipoprotein ProX. There were six mutations found in each of these genes, although no clear pattern relating to either phylogenetic or transmission status could be discerned, with mutations found in both transmission and non-transmission samples, as well as many samples having more than one of these mutations. The *nusG* (beta = 0.791, P = 5.8 × 10^–10^) encoded protein participates in transcription elongation, termination and anti-termination. There are five key mutations (S206G, E186A, R124L, A161V, F232C). By locating their position on a phylogenetic tree, only R124L was supported by isolates in more than one clade (S5 Figure). The *wag31* gene (beta = 0.912, P = 3 × 10^–7^) codes for a cell wall synthesis protein, but only one mutation (G67S) was associated with a single small transmission clade (n = 5) (S5 Figure). The *murA* gene codes for a peptidoglycan biosynthesis pathway, and had five mutations (E226K, R247L, D318A, H394Y, E414K), but none were found in more than one clade and only two mutations overlapped with transmission samples (H394Y, E226K) (S5 Figure).

**Figure 2:**
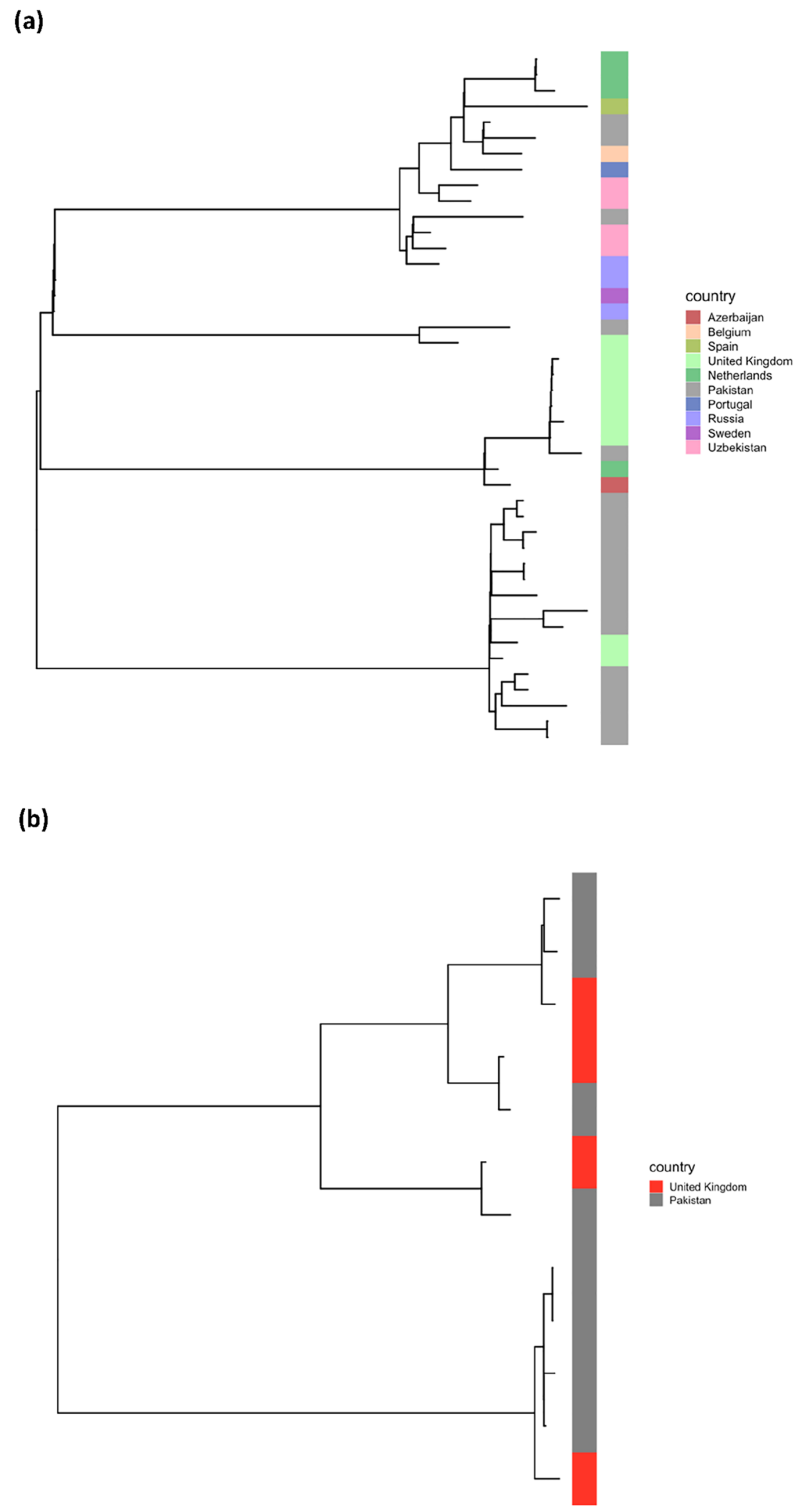
Phylogenetic trees for sub-lineages involving Pakistan samples and closely-related global isolates from previously published datasets. **(a)** Sub-lineage 2.2.1 (19 Pakistan, 25 other). **(b)** Lineage 3 (8 Pakistan, 4 UK).

The transmission clusters involved six main sub-lineages (1.1.2, 2.2.1, 3, 3.1.2, 4.5, 4.9), and we looked for similar isolates in other populations within the global 34 k dataset. Using a more relaxed cut-off of 20 SNPs difference to allow for greater time between transmission events, three of the sub-lineages (3, 2.2.1, 4.5) revealed similar isolates collected from other countries (Fig. 2). Lineage 2.2.1 had 19 Pakistan isolates linked to 29 global samples, mostly from countries in Europe and Central Asia. Lineage 3 had 8 Pakistan isolates linked to 5 other samples from the UK, while sub-lineage 4.5 had two Pakistan samples linked to a single isolate from the UK.

### Drug resistance mutations

The common mutations underlying genotypic drug resistance were in known loci. These included mutations in *rpoB* (D435GFYV 293/460, S450LFWY 308/460) linked to rifampicin, *katG* (S315NIT 374/416) and *fabG1* (−15C > T 52/416) linked to isoniazid, *embB* (G406ASDC 51/385, M306ILV 280/385, Q497RKP 40/385) linked to ethambutol, *gyrA* (A90V 68/277, S91P 22/277, D94GAHYN 195/277) linked to fluoroquinolones, and *pncA* (118 low frequency < 25/258) linked to pyrazinamide (Table 2). A high proportion of mutations detected were present in the global 34 k dataset, including *pncA* 93/118, *katG* 19/38, *rpoB* 37/39, and *embB* 21/21. Nearly half all mutations identified (156/313) were present in single isolates, of which the majority were in the 34 k dataset (101/156) and absent from sensitive strains (S7 Table).

**Table 2:** Number of samples with known drug resistance-associated mutations.

Drug	N	Gene	Change [N]
Aminoglycosides	129	*rrs*	1401a > g [74], 514a > t [3], 906a > g, [2], 1484g > t [1], 514a > c [47], 905c > g [2], 517c > t [8]
Capreomycin	3	*tlyA*	198_198del [1], N236K [2]
Cycloserine	2	*alr*	M343T [1], L113R [1]
Ethambutol	385	*embA*	−12C > T [19], −16C > G [2], −16C > T [12], −11C > A [5]
		*embB*	G406A [13], G406S [8], M306I [132], G406D [22], G406C [6], Q497R [20], Q497K [9], Q497P [2], Q853P [2], E405D [1], E504D [2], A313V [1], M306L [20], M306V [127], Y319C [1], Y319S [1], Y334H [2], S347I [1], D354A [7], D1024N [29], D328Y [3]
Ethionamide	54	*ethA*	1200_1201del [1], 1054_1054del [1], 599_599del [1], 1261_1262insCGAGC [1], 1018_1018del [1], 1047_1047del [1], 1300_1301insGT [1], 61_61del [1], 671_671del [1], 1290_1291insC [1], 4326936_4328449del [5], 4326943_4328449del [1], 4326944_4328449del [1], 4327038_4327099del [1], Q269* [4], Q347* [6], L272P [1], L397R [2], T61M [1], 672_673insG [2], 673_674insGC [1], 140_140del [3], 150_150del [1], 299_299del [3], 313_319del [1], 352_365del [2], 382_383insG [4], 392_392del [2], 404_405insAT [1], 703_703del [1], 755_756insGC [2], 825_825del [1]
Fluoroquinolones	277	*gyrA*	G88A [1], G88C [3], D89N [4], A90V [68], S91P [21], D94G [128], D94A [19], D94H [4], D94Y [17], D94N [24]
		*gyrB*	R446C [1], S447F [5], I486L [1], T500N [3], E501D [4]
Isoniazid	416	*katG*	22_23insA [1], 238_260del [1], 337_337del [1], 679_680insGC [1], 87_87del [1], 974_974del [1], 2148451_2164815del [1], 2149885_2172950del [1], 2151318_2157225del [1], 2152294_2157889del [1], A172V [1], R104Q [1], D259E [1], G297V [1], S140N [2], S315N [8], S315I [1], S315T [365], T275A [2], T380I [3], W191R [2], W328S [1], Y155C [1], Y155S [2], Y337C [1], Y413H [1], V1A [2], 1176_1177insG [1], 1196_1197insGA [1], 1284_1284del [1], 1328_1328del [1], 1486_1487insC [1], 2005_2006insG [2], 58_58del [1], 58_59insCT [1], 596_596del [1], 371_371del [1], 60_61insGT [1]
		*ahpC*	−54C > T [4], −81C > T [2]
Kanamycin	5	*eis*	−10G > A [1], −14C > T [3], −37G > T [1]
Pyrazinamide	258	*pncA*	−11A > C [4], −11A > G [15], −12 T > C [1], 108_108del [1], 13_14insGA [1], 166_167insG [1], 194_203del [1], 206_207insC [1], 209_210insACC [1], 226_236del [1], 230_231insA [1], 283_283del [1], 314_315insG [2], 346_347insC [2], 377_378insGA [1], 382_383insG [1], 391_392insG [2], 391_392insGG [17], 393_394insC [1], 408_409insT [1], 412_413insCATT [1], 417_418insG [3], 424_425insGA [2], 429_429del [1], 430_431insG [1], 438_439insCG [1], 455_456insATGGCTTGGC [2], 501_502insC [1], 53_53del [1], 61_62insG [1], 7_7del [1], 2285437_2291074del [1], 2288627_2289103del [2], 2288776_2288836del [1], 2288825_2289242del [1], 2289006_2290299del [1], A134V [1], A143D [1], A146V [2], A171T [4], A3E [2], R140G [4], D12A [3], D136Y [1], D49N [1], D49G [1], D63G [1], D63H [4], C14R [1], C72Y [1], Q10* [1], Q10R [5], Q10P [5], Q141P [4], G105D [1], G108R [1], G132S [3], G78S [4], G78V [1], G97S [3], H51Q [1], H57P [1], H57Y [4], H71R [3]. H71Y [3], H82R [1], I133T [2], I31S [1], I5T [2], I6M [1], I6T [1], L156P [1], L159R [2], L19R [2], L27P [1], L35P [1], L4S [2], L4W [1], L85P [1], K96R [2], K96E [1], K96T [2], M175T [2], M1T [1], F58L [1], F94L [1], P54L [23], P62L [2], P62S [2], P69R [3], S104R [1], S164P [1],S67P [4], T100I [1], T135P [4], T142A [1],T142M [1], T160P [3], T47P [3], T61P [1], T76I [2], T76P [6], W119R [3], W68* [1], W68R [2], W68C [3], W68G [2], W68S [1], Y103C [1], Y34S [1], Y41* [1], V128G [1], V139A [4], V139G [1], V180F [8], V45G [1], V7F [2], V9G [2]
Rifampicin	460	*rpoB*	1296_1297insTTC [2], 1306_1308del [3], A286V [2], N437Y [1], D435G [5], D435F [2], D435Y [30], D435V [32], Q429H [2], Q432H [1], Q432L [3], Q432K [4], Q432P [1], H445R [2], H445N [8], H445D [6], H445C [2], H445Q [2], H445L [9], H445P [2], H445Y [11], I480V [1], I491F [1], L430R [3], L430P [8], L452P [12], M434I [11], S428R [1], S428T [1], S441Q [1], S441L [2], S450L [293], S450F [2], S450W [9], S450Y [1], S493L [1], T400A [1], T444I [1], V170F [2]
		*rpoC*	D747A [1], G332R [6], I491T [13], I885V [1], L527V [5]
Streptomycin	172	*gid*	102_102del [2], 115_115del [3], 351_351del [4], 4407713_4407860del [1], A80P [3], L79S [1]
		*rpsL*	K43R [126], K88R [21], K88M [1], K88T [2]
INH, Ethionamide	58	*fabG1*	−15C > T [52], −17G > T [1], −8 T > A [1], −8 T > C [4]
		*inhA*	I194T [4], I21T[1], S94A [4], I21V [1]
PAS	10	*folC*	E153A [1], I43S [5], R49W [1], I43T [1]
		*thyX*	−16C > T [2]
BDQ, CFZ	1	*mmpR5*	192_193insG [1]

*BDQ* bedaquiline, *CFZ* clofazimine, *INH* isoniazid, *PAS* para aminosalicylic acid.

*Premature stop codon.

We investigated isolates that had a DST implying resistance, but no established genetic mutations to explain this phenotype. There were 82 isolates (100/2430 tests; (S2 Table)) with this discordance across 9 drugs (amikacin (9), capreomycin (2), ciprofloxacin (4), ethambutol (17), isoniazid (25), kanamycin (7)), pyrazinamide (24), rifampicin (6), streptomycin (6)). We identified 68 distinct genetic markers in candidate genes to potentially explain the discordance (Table 3). Twenty-nine (42.6%) mutations had strong evidence of being linked with drug resistance, including from functional consequences, homoplasy or global data information^7,14^. Forty-six (67.6%) mutations were present in the global 34 k dataset, and all of these were absent in sensitive strains (S8 Table), reinforcing them as putatively resistant related.

**Table 3:** Putative novel drug resistant mutations.

Drug	Gene	Change [N]
Amikacin	*rrs*	−92 T > G [1], 878 g > a [2]
Ciprofloxacin	*gyrA*	A288D [1]
	*gyrB*	−162C > CG [1], A432V [1]
Ethambutol	*embA*	−16C > A [2], −27TA > T [1], −42CAT > C [1], −8C > A [1], **P455Q** [1], **V534A** [1]
	*embB*	R524H [1], D328H [1], **D328F** [2], L172R [2], **F330L** [1], T546I [1]
	*ubiA*	G268D [1], **F238I** [1], **V188L** [1]
Isoniazid	*ahpC*	−52C > A [1], −72C > T [1], −76 T > A [4], −76 T > C [1], −**76 T > G** [1], −93G > A [1]
	*kasA*	M72I [1], **F402I** [1]
	*katG*	**587_588insGGT** [1], A122D [1], A348G [1], **R484G** [1], D189Y [1], Q36* [1]**,** **G186D** [1]**,** **G299D** [1], I103V [1], **L298S** [1]**,** M105I [1], F408S [1], P100T [1], T271I [1], T475I [1], T625K [1], W204* [1], W438* [1], **Y197D** [1]
Kanamycin	*eis*	L386I [1]
	*rrs*	−92 T > G [1]
Pyrazinamide	*pncA*	−7 T > G [1], **392_393insGGT** [1]**,** **451_462del** [1]**,** **511_512insTCGCCG** [1], L120R [4], P62T [1], P69T [1], S18* [1], V130M [1], V180A [1]
	*rpsA*	−**98A > T** [1]**,** **Q410R** [2]
Rifampicin	*rpoB*	**1291_1292insGCC** [1], 1294_1296del [1], 1309_1311del [1]
Streptomycin	*gid*	A119D [1], A82P [1], D67G [1], G71* [2]

*Based on absence in the curated TB-Profiler mutation list; bolded, if not observed in a large TB Global dataset (34 k^7^); underlined, if with multiple levels of evidence for drug resistance (see S8 Table).

For rifampicin resistance, we identified three inframe indels in *rpoB* (1291_1292insGCC, 1294_1296del and 1309_1311del) in three isolates. For isoniazid, several nonsense mutations in *katG* were found, with 3 mutations leading to premature stop codons (W438*, W204*, Q36*) and a frameshift mutation (587_588insGGT). For ethambutol resistance, variants in the *embA* promoter region (−42CAT > C, −27TA > T-16C > A, −8C > A) and *embB* were observed. For pyrazinamide resistance, several potentially new mutations were found in *pncA*, including three inframe indels (511_512insTCGCCG, 392_393insGGT and 451_462del), a premature stop codon (S18*), and SNPs in both the coding region (Val180Ala) and the promoter (−7 T > G). For streptomycin resistance, several mutations were found in *gid* including a premature stop codon (G71*), a frameshift (102_102del), and SNPs (A119D, A82P and D67G). These SNPs were found in the 34 k global dataset, and likely acquired as the result of homoplasy. The *gid* A119D mutation was present in 15 isolates (ten different sublineages), of which two had DSTs that reported resistance. The *gid* A82P mutation was present in three isolates from two different sub-lineages, but no DST data was available for these samples. The *gid* D67G was present in 38 global isolates from five different sublineages. Of these, seven isolates had DST data available with four presenting with resistance.

For second line injectables, the *rrs* 878g > a mutation (seen previously^2^) was observed in four lineage 3 strains with three independent homoplastic acquisitions, indicating it is unlikely to be strain-specifc. Mutations in *rrs* are generally clustered in two regions with the most common mutations involved with streptomycin resistance being located around position 514 and those involved with resistance to amikacin, kanamycin and capreomycin located around 1401. The *rrs* 878g > a falls between the two mutation hotspots, and of the three strains which had DST data (amikacin and kanamycin) in this study, two were resistant to both amikacin and kanamycin and the other was sensitive to both. For fluoroquinolones, the *gyrA* A288D mutation was found in three lineage 3 isolates and was acquired in each sample independently. One isolate tested resistant to ciprofloxacin with no known resistance mutation found in the *gyrA* and *gyrB* genes.

## Discussion

The use of whole genome sequencing as a diagnostic is gaining traction in low resource and high TB burden settings, where it has the potential to have greater public health impact^5,7,15^. Portable sequencing platforms and multiplexing of *M. tuberculosis* isolates are making the application of WGS, both timely and cost effective^5^. Our findings in the largest analysis of isolates from Pakistan to date revealed that lineage 2 and 4 strains, which are pre-XDR and XDR-TB, are potentially being transmitted in the country. Evidence of increased transmission among lineages 2 and 4 is consistent with previous characterisations of these clades as more transmissible^7^, and therefore their strain-types should be monitored more closely despite greater prevalence of lineage 3. It is surprising that pre-XDR and XDR-TB samples were found to be clustered more than expected compared to MDR-TB isolates given the usual fitness cost of drug resistance. This observation suggests that compensatory mutations ought to be investigated in future work. Similarly, the finding that mutations in *nusG, Rv2307B, wag31, proX* and *murA* genes maybe associated with transmission should be followed-up experimentally, where those with variants appearing in more than one clade could be priortised. Advances in the characterisation of transmission events^16^, GWAS^9,17^ and machine learning methods^18,19^ could enhance the ability to detect mutations linked to transmissiblility. However, host factors and host–pathogen genetic interactions are also likely to be important. More broadly, the routine collection, processing and WGS of *M. tuberculosis* DNA across Pakistan will provide robust insights into mutations underlying drug resistance and geo-temporal dynamics.

Whilst our study uses a convenience sample that is not necessarily representative of the proportions of MDR-TB in the wider Pakistan population, it is enriched by the presence of many mutations that lead to drug resistance. The enrichment of drug resistant isolates from endemic TB regions with high transmission will reveal important resistance mutations, including potential novel variants. To investigate the underlying mechanisms of drug resistance, we compared susceptibility profiles from phenotypic methods and genotypic prediction. This analysis led to the identification of a number of potential new drug resistance mutations, including in genes causing resistance to rifampicin, isoniazid, ethambutol and pyrazinamide. Three inframe deletions were found in the rifampicin resistance determining region of *rpoB*. Inframe deletions have not been widely reported as a major mechanism of resistance to rifampicin and it is surprising to see a relatively high number of these mutations in our dataset. Previously unreported nonsense mutations were also found in the *katG* gene, a locus responsible for resistance to isoniazid. A novel nonsense mutation, frameshift and inframe indels were found in the *pncA* gene, which codes for the activator of pyrazinamide. Mutations in the promoter region of the *pncA* gene lead to changes in the expression of PncA and resistance^20^. The identified −7 T > G promoter mutation is thus likely to cause resistance. However the functional effects of SNPs found in the coding region of *pncA* are more difficult to predict^20^. The *pncA* V180A mutation has been reported previously to be associated with pyrazinamide resistance^20^. For streptomycin, we observed several point mutations and a premature stop codon in the *gid* gene. The *gid* D67G mutation was found in 38 isolates in the 34 k global dataset^7^, of which 57% of those were phenotypically resistant to streptomycin. The incomplete penetrance of the streptomycin-associated *gid* D67G mutation could be explained by the relative low-level resistance conferred by mutations in *gid,* which could be below established critical cut-offs of minimum inhibitory concentration for susceptibility phenotyping, but still elevated with respect to wild-type.

Overall, our work reinforces that the adoption of WGS platforms as a diagnostic tool, combined with mutational databases of drug resistance markers, will inform clinical decision making. The ability to perform WGS for genomic investigations across time and geography will improve the understanding of transmission dynamics, and inform control programmes to reduce disease burden. The benefits will be greatest in high prevalence TB settings, typically low and middle income countries, such as Pakistan. Although WGS is not currently at a viable level of affordability, it is anticipated that amplicon and whole genome approaches using (portable) next generation platforms will shortly become simple, affordable and accessible rapid diagnostics compared to traditional laboratory-based methods that currently require specialist training, equipment and long culture times. Importantly, there is evidence that WGS is more detailed and accurate in its profiling of drug resistance than traditional DST, thereby likely to improve treatment and mortality outcomes in drug-resistant TB in high-burden countries^21^.

## Methods

### Sequence data and processing

WGS were sourced across six studies^2,9–13^ (ENA accessions: PRJEB7798, PRJEB10385, PRJEB25972, PRJEB32684, PRJEB43284), where contributing isolates belong to a single patient. Phenotypic DSTs were conducted using WHO endorsed methods, as specified in descriptions of the original studies^2,9–13^. Raw reads were trimmed to remove low-quality sequences in Trimmomatic (v0.39)^22^, and aligned to the H37Rv reference genome (AL123456) with BWA mem (v0.7.17)^23^. SNPs and indels called by samtools software^24^ were processed using gatk GenotypeGVCFs (v4.1.3.0) (gatk.broadinstitute.org). Monomorphic SNPs and variants in non-unique regions of the genome (e.g. *pe/ppe* genes) were excluded. A multi-FASTA format file was created from the filtered SNP file and H37Rv reference fasta using bedtools makewindows (v2.28.0)^25^. This multiple alignment was used to construct a phylogenetic tree with IQ-TREE (v1.6.12), involving a general time reversible model with rate heterogeneity set to a discrete Gamma model and an ascertainment bias correction (parameters −m GTR + G + ASC), with 1000 bootstrap samples^26^. Pairwise distance matrices were calculated in Plink software (v1.90b4)^27^. Drug resistance and lineages were predicted in silico from raw sequence data using TB-Profiler (v2.4)^5^. The Pakistan analysis results were compared to a global collection of 34 k *M. tuberculosis* with WGS and DST data^7^.

A cut-off of 10 SNPs difference was established to define transmission clades, and label samples as “transmitted” or “non-transmitted”. A sensitivity analysis was performed to assess the impact of changing the cut-off. Linear mixed models were used perform a GWAS of transmissibility using SNPs, location, drug resistance and adjusting for *M. tuberculosis* (sub-)lineage and outbreak-based population structure, being implemented in GEMMA (v.1.1.2) (http://www.xzlab.org/software.html). We report association p-values less than a Bonferroni cut-off based on testing 4,000 genes (P < 1.25 × 10^–5^). To identify if samples involved in transmission clades (> 10 samples) were similar to others (< 20 SNPs) in the global dataset (n = 34 k)^7^, we constructed phylogenetic trees using FastTree for the relevant sub-lineages (1.1.2, 2.2.1, 3, 3.1.2, 4.5, 4.9). The likelihoods of ancestral locations were inferred with the ape (v5.0) and phytools packages in R.

## Supplementary Information

Supplementary Table S7.

Supplementary Table S8.

Supplementary Information.

## References

[1] World Health Organization (WHO). *Global Tuberculosis Report 2021*. (2021).

[2] Jabbar A (2019). Whole genome sequencing of drug resistant Mycobacterium tuberculosis isolates from a high burden tuberculosis region of North West Pakistan. Sci. Rep..

[3] World Health Organisation. *Meeting Report of the WHO Expert Consultation on Drug-Resistant Tuberculosis Treatment Outcome Definitions, 17–19 November 2020*. (2020).

[4] Phelan J (2016). The variability and reproducibility of whole genome sequencing technology for detecting resistance to anti-tuberculous drugs. Genome Med..

[5] Phelan JE (2019). Integrating informatics tools and portable sequencing technology for rapid detection of resistance to anti-tuberculous drugs. Genome Med..

[6] Coll F (2015). Rapid determination of anti-tuberculosis drug resistance from whole-genome sequences. Genome Med..

[7] Napier G (2020). Robust barcoding and identification of *Mycobacterium tuberculosis* lineages for epidemiological and clinical studies. Genome Med..

[8] Glynn JR (2015). Whole genome sequencing shows a low proportion of tuberculosis disease is attributable to known close contacts in rural Malawi. PLoS ONE.

[9] Coll F (2018). Genome-wide analysis of multi- and extensively drug-resistant *Mycobacterium tuberculosis*. Nat. Genet..

[10] Kanji A (2016). Alternate efflux pump mechanism may contribute to drug resistance in extensively drug-resistant isolates of *Mycobacterium tuberculosis*. Int. J. Mycobacteriol..

[11] Ali A (2015). Whole genome sequencing based characterization of extensively drug-resistant mycobacterium tuberculosis isolates from pakistan. PLoS ONE.

[12] Cryptic-Consortium. Prediction of susceptibility to first-line tuberculosis drugs by DNA sequencing. *N. Engl. J. Med.* **379**, 1403–1415 (2018). 10.1056/NEJMoa1800474 PMC6121966 30280646

[13] Khan AS (2021). Characterization of rifampicin-resistant *Mycobacterium tuberculosis* in Khyber Pakhtunkhwa, Pakistan. Sci. Rep..

[14] Phelan JE (2019). Integrating informatics tools and portable sequencing technology for rapid detection of resistance to anti-tuberculous drugs. Genome Med..

[15] Phelan J (2016). *Mycobacterium tuberculosis* whole genome sequencing and protein structure modelling provides insights into anti-tuberculosis drug resistance. BMC Med..

[16] Sobkowiak B (2020). Bayesian reconstruction of mycobacterium tuberculosis transmission networks in a high incidence area over two decades in Malawi reveals associated risk factors and genomic variants. Microb. Genomics.

[17] Oppong YEA (2019). Genome-wide analysis of *Mycobacterium tuberculosis* polymorphisms reveals lineage-specific associations with drug resistance. BMC Genomics.

[18] Libiseller-Egger J, Phelan J, Campino S, Mohareb F, Clark TG (2020). Robust detection of point mutations involved in multidrug-resistant Mycobacterium tuberculosis in the presence of co-occurrent resistance markers. PLoS Comput. Biol..

[19] Deelder W (2019). Machine learning predicts accurately *Mycobacterium tuberculosis* drug resistance from whole genome sequencing data. Front. Genet..

[20] Tunstall, T., Phelan, J., Eccleston, C., Clark, T. G. & Furnham, N. Structural and genomic insights into pyrazinamide resistance in *Mycobacterium tuberculosis* underlie differences between ancient and modern lineages. *Front. Mol. Biosci.* **8** (2021). 10.3389/fmolb.2021.619403 PMC8372558 34422898

[21] Zürcher K (2021). Mortality from drug-resistant tuberculosis in high-burden countries comparing routine drug susceptibility testing with whole-genome sequencing: A multicentre cohort study. Lancet Microbe.

[22] Bolger AM, Lohse M, Usadel B (2014). Trimmomatic: A flexible trimmer for Illumina sequence data. Bioinformatics.

[23] Li, H. *Aligning Sequence Reads, Clone Sequences and Assembly Contigs with BWA-MEM*. (2013).

[24] Li H (2011). A statistical framework for SNP calling, mutation discovery, association mapping and population genetical parameter estimation from sequencing data. Bioinformatics.

[25] Quinlan AR, Hall IM (2010). BEDTools: A flexible suite of utilities for comparing genomic features. Bioinformatics.

[26] Nguyen L-T, Schmidt HA, von Haeseler A, Minh BQ (2015). IQ-TREE: A fast and effective stochastic algorithm for estimating maximum-likelihood phylogenies. Mol. Biol. Evol..

[27] Purcell S (2007). PLINK: A tool set for whole-genome association and population-based linkage analyses. Am. J. Hum. Genet..

