# Supplementary material for: Characterisation of drug-resistant Mycobacterium tuberculosis mutations and transmission in Pakistan
Source: Sci Rep. 2022 May 11;12:7703. doi: 10.1038/s41598-022-11795-4 (PMC9095715; doi:10.1038/s41598-022-11795-4)

S1 Table: Strain-types

Lineage	Large sequence polymorphisms lineage	Spoligotype family	Region of difference no.	N	%
3	East-African-Indian	CAS	750	363	67.9
2.2.1	East-Asian (Beijing)	Beijing-RD181	105;207;181	29	5.4
4.5	Euro-American	H;T	122	23	4.3
4.9	Euro-American (H37Rv-like)	T1	None	22	4.1
1.1.2	Indo-Oceanic	EAI3;EAI5	239	14	2.6
3.1.2	East-African-Indian	CAS;CAS2	750	11	2.1
3.1.2.1	East-African-Indian	CAS2	750	10	1.9
3.1.3	East-African-Indian	CAS	750	9	1.7
4.8	Euro-American (mainly T)	T1;T2;T3;T5	219	9	1.7
1.2.2.2	Indo-Oceanic	NA	239	7	1.3
4.2.2.2	Euro-American (Ural)	T;LAM7-TUR	None	7	1.3
2.2.1.1	East-Asian (Beijing)	Beijing-RD150	105;207;181;150	5	0.9
3.1	East-African-Indian	Non-CAS1-Delhi	750	4	0.7
2.2.1.2	East-Asian (Beijing)	Beijing-RD142	105;207;181;142	2	0.4
4	Euro-American	LAM;T;S;X;H	None	2	0.4
4.1.1.1	Euro-American (X-type)	X2	183	2	0.4
4.1.1.3	Euro-American (X-type)	X1;X3	193	2	0.4
4.2.1	Euro-American (TUR)	H3;H4	None	2	0.4
4.2.2	Euro-American (Ural)	T;LAM7-TUR	None	2	0.4
4.6	Euro-American	T;LAM	None	2	0.4
4.6.5	Euro-American	T;LAM	None	2	0.4
1.1.3.3	Indo-Oceanic	NA	239	1	0.2
4.1.1.2	Euro-American (X-type)	X1	None	1	0.2
4.1.2.1	Euro-American (Haarlem)	T1;H1	182	1	0.2
4.6.2	Euro-American	T;LAM	726	1	0.2
4.6.2.1	Euro-American	T3	726	1	0.2
4.6.2.2	Euro-American (Cameroon)	LAM10-CAM	726	1	0.2

S2 Table: Drug-resistant samples according to drug susceptibility tests (DSTs) and genotypic predictions

Drug	DST N	DST resistant N	DST resistant %	Genotypic resistant* N	Genotypic resistant %	DST Susceptible Genotypic resistant	DST resistant Genotypic non-resistant
Rifampicin	487	417	85.6	460	86.0	6	6
Isoniazid	487	411	84.4	435	81.3	7	25
Ethambutol	479	265	55.3	385	72.0	96	17
Pyrazinamide	444	189	42.6	258	48.2	42	24
Streptomycin	43	24	55.8	238	44.5	4	6
Ofloxacin	85	46	54.1	277	51.8	5	0
Moxifloxacin	52	4	7.7	277	51.8	29	0
Levofloxacin	0	-	-	277	51.8	0	0
Amikacin	110	42	38.2	75	14.0	0	9
Kanamycin	112	44	39.3	79	14.8	0	7
Capreomycin	57	15	26.3	78	14.6	18	2
Ciprofloxacin	37	37	100	277	51.8	0	4
Ethionamide	37	6	16.2	102	19.1	11	0
PAS	0	-	-	10	1.9	0	0
Cycloserine	0	-	-	2	0.4	0	0
Clofazimine	0	-	-	1	0.2	0	0
Bedaquiline	0	-	-	1	0.2	0	0
Linezolid	0	-	-	0	0.0	0	0
Delamanid	0	-	-	0	0.0	0	0
Fluoroquinolones	174	87	50.0	277	51.8	-	-
Aminoglycosides	322	125	38.8	75	14.0	-	-

* from TB-Profiler; PAS para aminosalicylic acid; DST drug susceptibility test

S3 Table: Drug resistance (DR) categories by Lineage (L)

DR status	L1 N	L1 %	L2 N	L2 %	L3 N	L3 %	L4 N	L4 %	Total N	Total %
Sensitive	1	4.5	0	0.0	53	13.4	6	7.5	60	11.2
Pre-MDR	2	9.1	0	0.0	28	7.1	1	1.3	31	5.8
MDR	12	54.5	25	69.4	242	61.0	49	61.3	328	61.3
Pre-XDR	0	0.0	7	19.4	25	6.3	15	18.8	47	8.8
XDR	5	22.7	4	11.1	48	12.1	9	11.3	66	12.3
Other	2	9.1	0	0.0	1	0.3	0	0.0	3	0.6
Total	22	100	36	100	397	100	80	100	535	100.0

MDR multidrug resistant, XDR extensively drug resistant

S4 Table: Sensitivity analysis of the clustering by SNP distance

SNP distance	No. Clusters	N	Median (Maximum)	L1	L2	L3	L4	Sensitive	Pre MDR	MDR	Pre XDR	XDR	Other DR
0	28	60	2 (3)	2	6	35	17	2	2	17	19	20	0
1	28	60	2 (3)	2	6	35	17	2	2	17	19	20	0
5	49	136	2 (17)	7	16	77	36	2	3	60	29	40	2
10	55	169	2 (22)	7	21	98	43	2	3	87	31	44	2
15	54	176	2 (22)	8	21	103	44	2	4	90	32	46	2
20	63	200	2 (22)	8	24	121	47	2	5	106	35	49	3
25	68	213	2 (22)	8	24	131	50	2	5	118	35	50	3
30	71	220	2 (22)	8	25	137	50	2	5	124	35	51	3

L Lineage, MDR multidrug resistant, XDR extensively drug resistant, DR drug resistance

S5 Table: Characteristics of 169 *M. tuberculosis* isolates in 55 clusters with a SNP distance of 10 compared to others

Characteristic		Trans. (169)	%	Non-trans. (366)	%	Odds ratio	95% CI	P-value
Lineage	1	7	4.1	15	4.1	1.00	-	-
	2	21	12.4	15	4.1	3.00	0.98 - 9.15	0.054
	3	98	58.0	299	81.7	0.70	0.28 - 1.77	0.454
	4	43	25.4	37	10.1	2.49	0.92 - 6.76	0.073
DR status	Sensitive/MDR	94	55.6	328	89.6	1.00	-	-
	Pre-XDR/XDR	75	44.4	38	10.4	5.79	3.67 - 9.14	$4.6x10^{-14}$
Location	Dera Ismail Khan	13	7.7	12	3.3	1.00	-	-
	Peshawar	46	27.2	31	8.5	1.37	0.55 - 3.39	0.497
	Other	30	17.8	31	8.5	0.89	0.35 - 2.27	0.812

Trans. transmitted

S6 Table: Genome-wide association analysis of transmission

Gene	Function	beta	95% CI	P-value
nusG	Transcription termination protein NusG	0.791	0.545 - 1.036	$5.8x10^{-10}$
Rv2307B	Hypothetical glycine rich protein	0.745	0.491 - 0.999	$1.5x10^{-8}$
wag31	Cell wall synthesis protein Wag31	0.912	0.567 - 1.256	$3.1x10^{-7}$
proX	Possible osmoprotectant binding lipoprotein ProX	0.706	0.423 - 0.988	$1.3x10^{-6}$
murA	Peptidoglycan biosynthesis pathway	0.660	0.380 - 0.939	$4.7x10^{-6}$

S7 Table: Number of samples with known drug resistance-associated mutations

S7_table_known_mutations.xls

S8 Table: Phenotypically resistance samples (n=82) with variants previously unknown to be associated with drug resistance.

S8_table_novel_mutations.xlsx

S1 Figure: Phylogenetic tree for the 535 *M. tuberculosis* isolates with individual genomic drug resistance predictions.

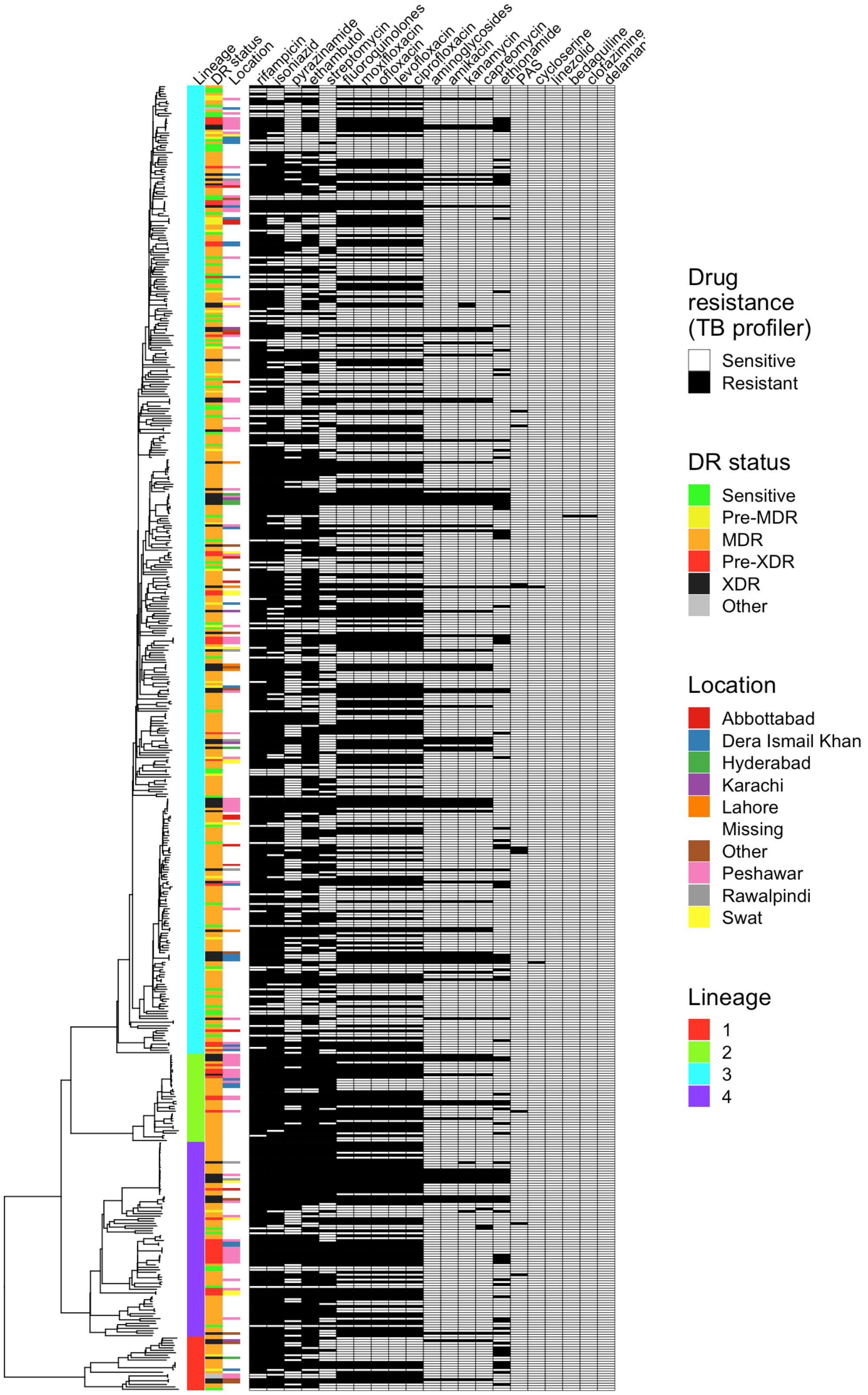

S2 Figure: SNP distance analyses and clusters (n=535). (top) Density of pairwise SNP differences for all samples; (bottom) number of clustering samples at minimum pairwise SNP difference thresholds

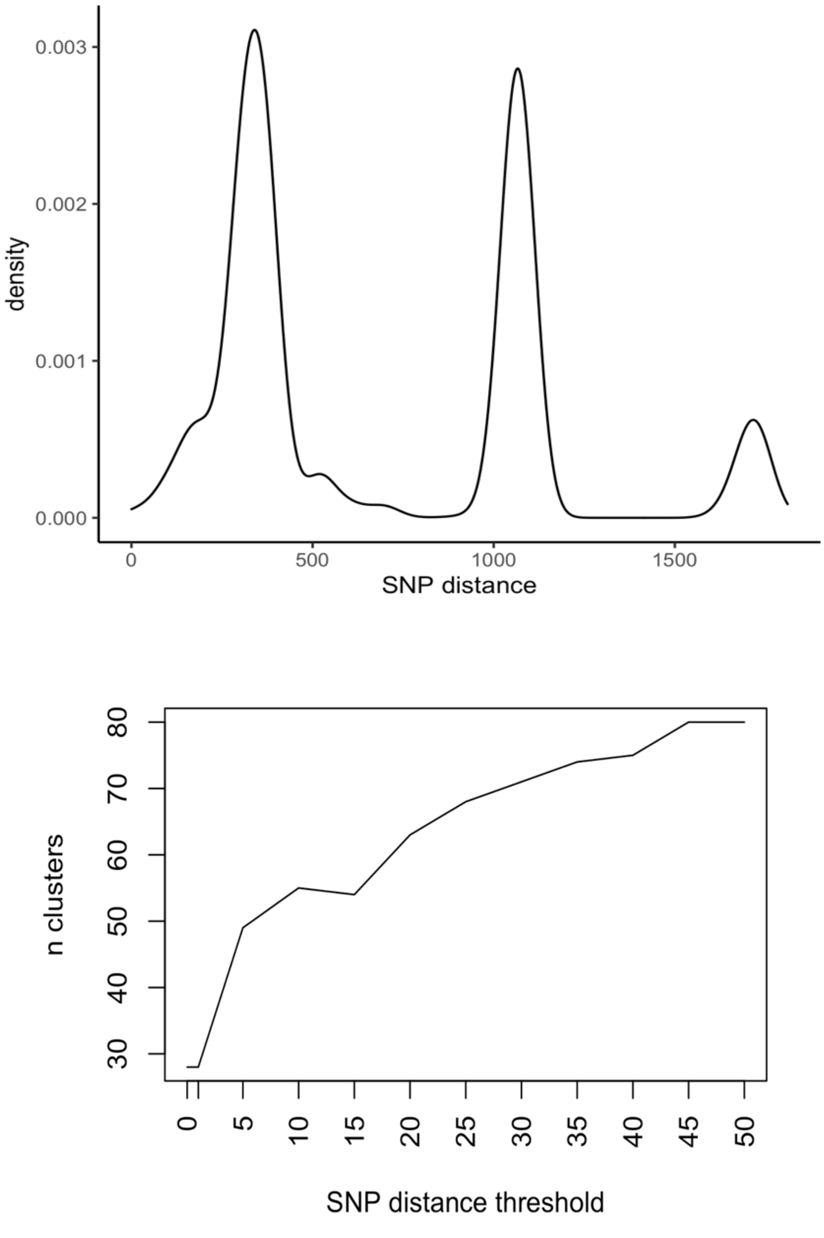

S3 Figure: Locations of samples in the transmission chains (n = 169)

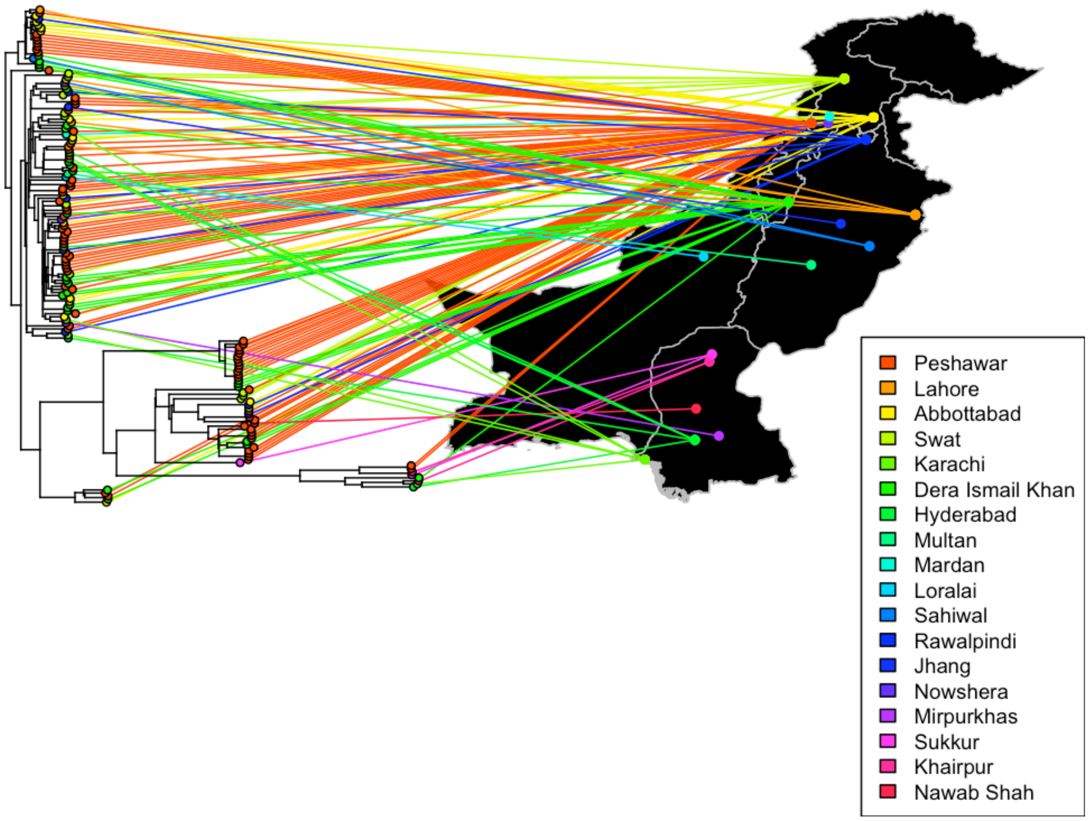

S4 Figure: The clusters with isolates at <= 10 SNP distance (n = 169), by lineage (top), drug resistance status (middle), and location (bottom).

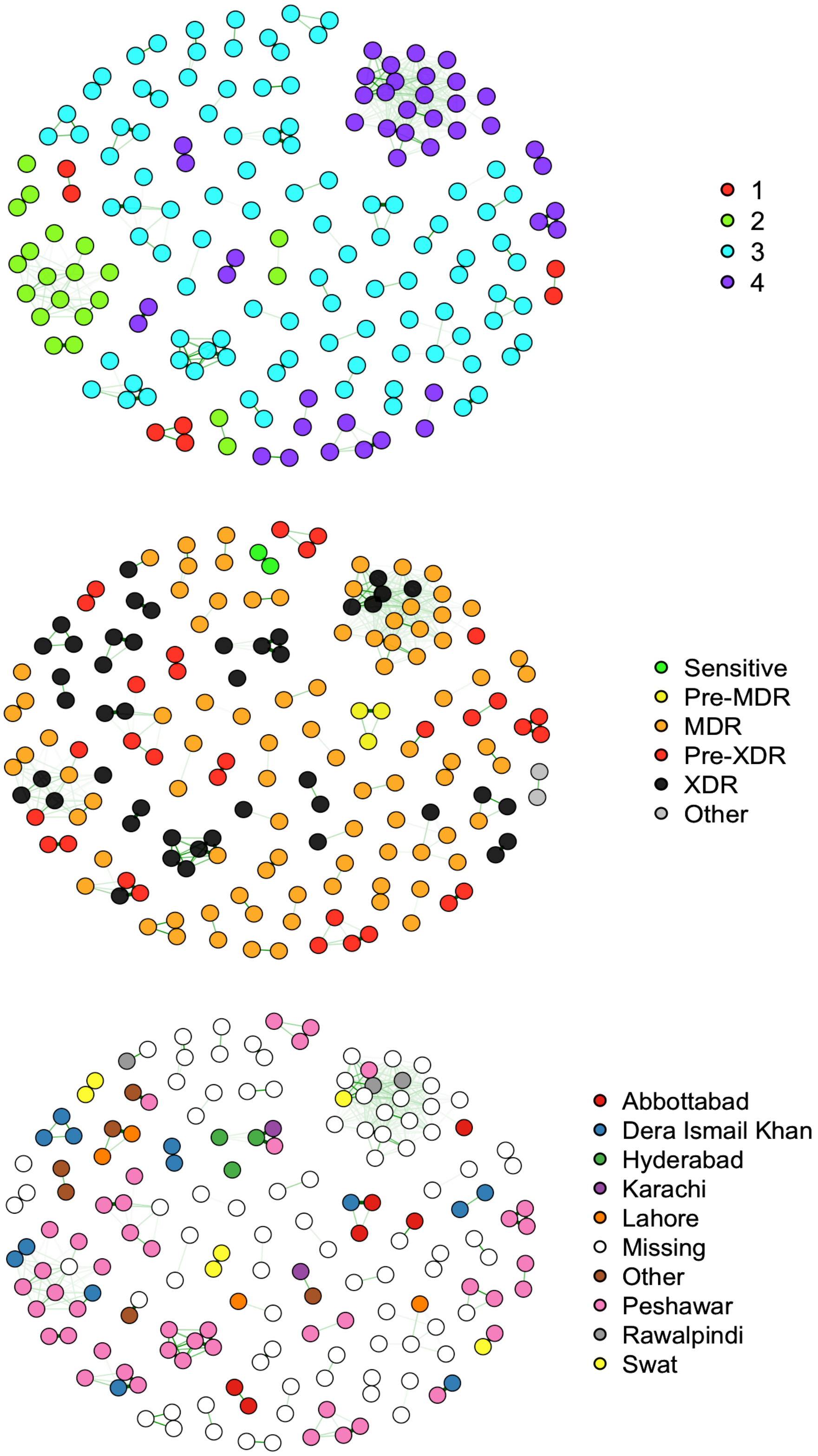

S5 Figure

Phylogenetic location of mutations in genes compared with location of transmission samples.

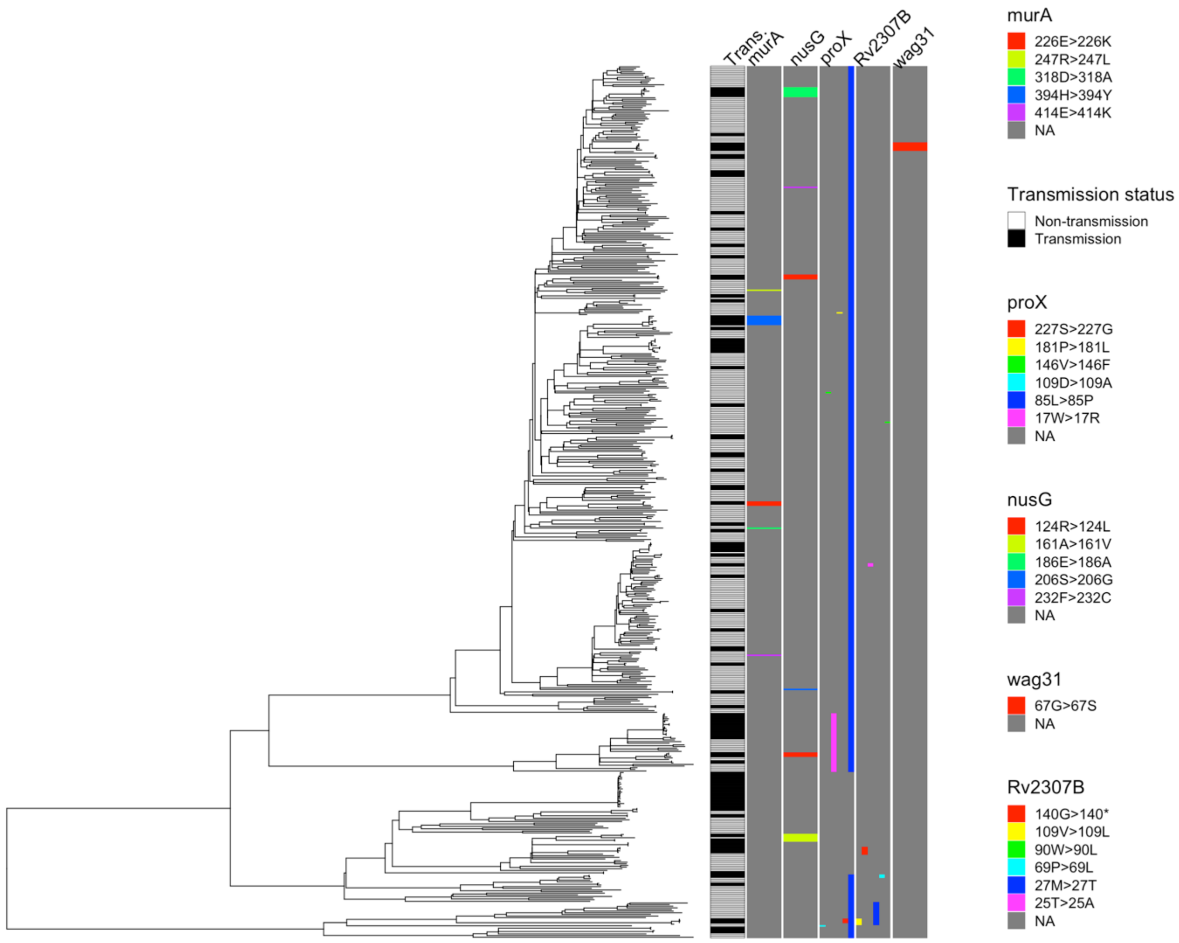

Supplement: Supplementary file 3 — Supplementary Information. [file 41598_2022_11795_MOESM3_ESM.pdf]
